# Lasing by driven atoms-cavity system in collective strong coupling regime

**DOI:** 10.1038/s41598-017-11799-5

**Published:** 2017-09-12

**Authors:** Rahul Sawant, S. A. Rangwala

**Affiliations:** Light and Matter Physics Group, Raman Research Institute, Sadashivanagar, Bangalore 560080 India

## Abstract

The interaction of laser cooled atoms with resonant light is determined by the natural linewidth of the excited state. An optical cavity is another optically resonant system where the loss from the cavity determines the resonant optical response of the system. The near resonant combination of an optical Fabry-Pérot cavity with laser cooled and trapped atoms couples two distinct optical resonators via light and has great potential for precision measurements and the creation of versatile quantum optics systems. Here we show how driven magneto-optically trapped atoms in collective strong coupling regime with the cavity leads to lasing at a frequency red detuned from the atomic transition. Lasing is demonstrated experimentally by the observation of a lasing threshold accompanied by polarization and spatial mode purity, and line-narrowing in the outcoupled light. Spontaneous emission into the cavity mode by the driven atoms stimulates lasing action, which is capable of operating as a continuous wave laser in steady state, without a seed laser. The system is modeled theoretically, and qualitative agreement with experimentally observed lasing is seen. Our result opens up a range of new measurement possibilities with this system.

## Introduction

While both cold atoms^1, 2^ and cavity physics^3, 4^ are central to atomic physics, few experiments combine an intra-cavity ensemble of cold atoms with a cavity^5–13^. Cooled and trapped atoms are spatially localized, natural linewidth limited and have high local densities, which allows spectral and spatial overlap with cavity modes. When a large number of these atoms are contained within the cavity mode volume, and the atom-cavity system is brought into resonance, collective strong coupling of atoms and light alters the transmission properties of resonant probe light through the cavity^3, 7, 12, 14–18^. This manifests in a change from a single transmission peak per spatial electromagnetic (EM) mode with a Lorentzian lineshape for an empty cavity, to zero transmission of the probe on atomic resonance. Further, on scanning the probe light around the atomic transition, symmetric red and blue detuned transmission peaks are observed. The frequency difference between the transmitted peaks is $2{g}_{0}\sqrt{{N}_{c}}$, where *g*_0_ is the single atom-cavity coupling and *N*_*c*_ is the effective number of atoms coupled to the cavity mode.

In this experiment, we trap within the cavity mode, fluorescing and continuously driven MOT atoms. The atom numbers in the present experiment are such that the collective strong coupling regime of atom-cavity mode interaction is accessible. We then pose the question, what is the effect of externally driven atoms on the coupled atom-cavity system? The answer is surprisingly different from the non-driven system. On scanning the cavity length, the system now emits light out of the cavity at red and blue detuning of the cavity with respect to the atomic resonance frequency. Since the magneto-optical trap (MOT) lasers are also the drive lasers and are red detuned, the experimentally broken symmetry reflects in the emission of light from the cavity atoms in a dramatic manner, where the red detuned light emitted is a laser and the blue detuned light is not a laser. Evidence of lasing for the red detuning is obtained from the series of experiments discussed below. The lasing here is not due to population inversion in the gain medium but due to multiphoton processes. The lasing is seeded by spontaneous emission from the driven atoms and not by an external seed/probe laser. When this system is probed with a probe laser coupled to the cavity, Fano-like resonances and line narrowing are seen for the probe, which is much narrower than all the line widths that are natural to the system. All the observations put together provide irrefutable evidence of lasing by the driven atom-cavity system.

In recent times, understanding of lasing with different gain mechanisms^10, 19–27^ has accelerated. For example, one such process relies on quantum interference between probability amplitudes in multilevel atoms and does not require population inversion^19, 20^. Raman processes between different hyperfine levels and its sublevels^10, 21, 22^, and levels involving external degrees of freedom^23, 24^ have been exploited as gain mechanisms including laser involving a single atom^25^. Much progress in understanding the role of collective effects on lasing^24, 26, 27^ has been made. William *et al*.^10^ showed lasing using three different gain mechanisms, Mollow gain, Raman transition between Zeeman sublevels and gain due to four-wave mixing, all of which were achieved with the same gain medium. Raman transition between Zeeman sublevels as a gain process was used by the same group to demonstrate random laser without any cavity and seed laser^28^. In the experiments here we demonstrate that continuous lasing seeded by spontaneous emission from the driven, non-inverted population of atoms in the cavity mode is possible and robust under reasonable experimental conditions. The gain mechanism for the lasing action is a result of multiphoton scattering^29, 30^. However, unlike previous works^10, 30^, we observe lasing action even when the cavity is not driven by a seed laser. Lezama *et al*.^31^ have earlier seen lasing in an atomic beam experiment, in a regime where atom-cavity coupling was very small for any co-operative effect to manifest.

## Results

### The experiment

**Figure 1:**
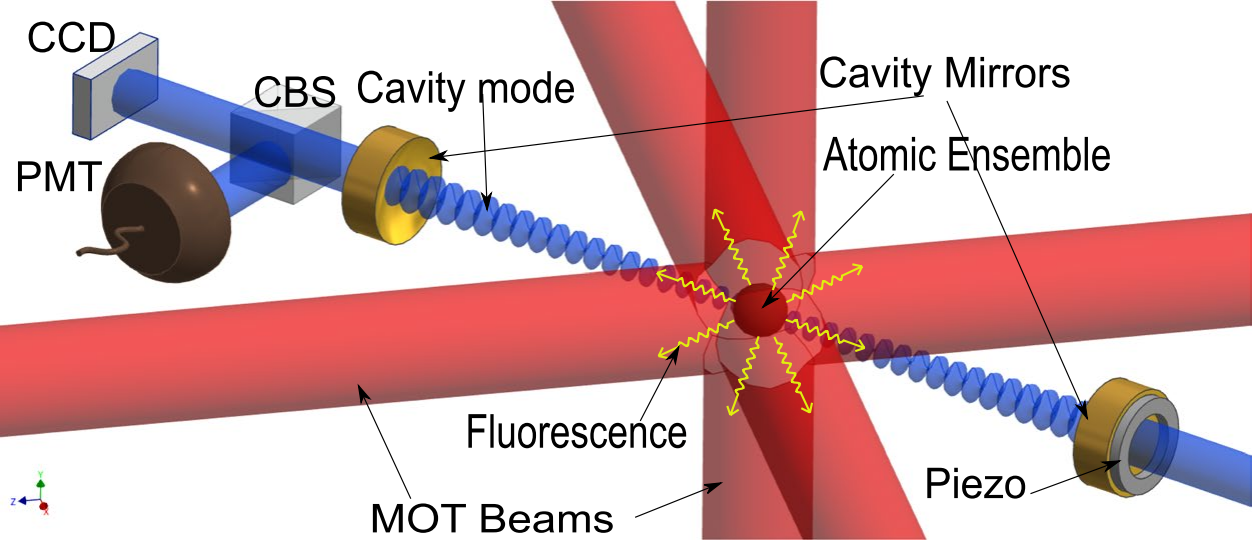
Experimental schematics. ^85^Rb atoms are cooled by the six red MOT beams and trapped with the help of magnetic gradient. The center of MOT beams and center of the cavity formed by the two mirrors overlap. PMT - Photomultiplier tube, CBS - Common beam splitter, CCD- Charge-coupled device (camera).

**Figure 2:**
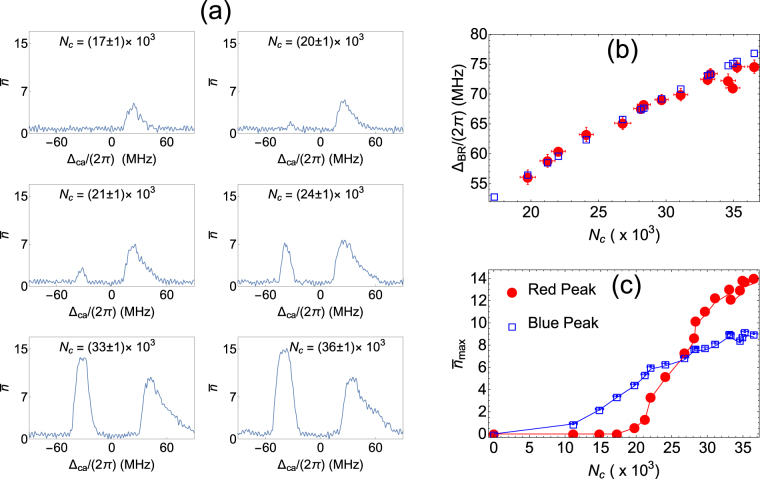
Change in emission from the cavity when the atom number coupled to the cavity is varied. (**a**) These graphs show cavity emission spectrum for different atom numbers. ${{\rm{\Delta }}}_{ca}={\omega }_{c}-{\omega }_{a}$ is detuning of empty cavity resonance from atomic transition and $\bar{n}$ is average intracavity photon number. The spatial mode for all the measurements is TEM_00_. (**b**) Red dots with error bars show the change in peak separation of the cavity emission with *N*_*c*_. Blue empty squares are corresponding calculated VRS ($2{g}_{0}\sqrt{{N}_{c}}$) values. ${{\rm{\Delta }}}_{BR}\mathrm{/(2}\pi )$–The frequency separation between the maximum of red and blue detuned peaks (**c**) Variation in the height of red and blue detuned peaks. In the range of measurement, the red peak shows a threshold behavior with atom number whereas the blue peak does not. ${\bar{n}}_{max}$–Maximum intracavity photon number for the red and blue detuned peaks. Total intensity of all cooling laser beams is 36 mW/cm^2^ for all the measurements. Here, the atom number is varied by changing the pressure of background atoms from which the ultracold atoms are loaded by laser cooling using the MOT beams. This is done by changing the intensity of deep blue LED’s mounted on the vacuum system. Blue wavelengths are very efficient at desorbing alkali atoms from the glass viewports and result in changes in vapor pressure. The detuning ${{\rm{\Delta }}}_{ca}\mathrm{/(2}\pi )$ is calibrated by monitoring the transmission of a laser beam which is frequency stabilized to the atomic transition through the cavity. For this, the scanning parameters are kept the same and there are no atoms in the cavity. The calibration between the voltage applied to the PZT and shift in frequency of the cavity resonance which is scanned is achieved by measuring the spectrum of a frequency stabilized laser with 20 MHz sidebands using the experimental cavity scan.

The experimental schematic is as shown in the Fig. 1, ^85^Rb atoms are cooled and trapped in a Magneto-optical trap (MOT). The center of the MOT and center of the enclosing Fabry-Pérot cavity are overlapped^12, 32^. The atomic ensemble is constantly illuminated by the six MOT light beams of 10 mm diameter and frequency red detuned by 13 MHz from the atomic transition (3-4′ transition of D2 line^33^). The magnetic field gradient of the MOT is 22 G/cm. No probe light is incident on the cavity mirrors unless specifically mentioned. The atom-cavity is in collective strong coupling regime, i.e., ${g}_{0}\sqrt{{N}_{c}} > {\rm{\Gamma }},\kappa$ ^3, 34^. Here *g*_0_/(2π) = 201 kHz is maximum single atom coupling strength, ${\rm{\Gamma }}\mathrm{/(2}\pi )=\mathrm{(6.06)}$ MHz is atomic exited state decay rate, 2*κ*/(2*π*) = 9.5±0.1 MHz is cavity full width at half maximum (FWHM), and *N*_*c*_ is an *effective number of atoms coupled to the cavity* which can be computed using overlap integral of the cavity mode and the observed atomic density profile. The free spectral range of the cavity is 3.28 GHz, the waist size of TEM_00_ cavity mode is 78 *μ*m, and the spacing between the TEM modes is 180 MHz. Using an annular piezoelectric transducer (PZT) attached to one cavity mirror, the length of the cavity and hence its resonance frequency (*ω*_*c*_) can be tuned. On scanning *ω*_*c*_ around the atomic transition, with frequency *ω*_*a*_ (3–4′ transition of D2 line^33^, which is also the cooling transition for the MOT), two peaks are observed in cavity emission. The cavity emission is monitored using a Photo Multiplier Tube (PMT) and a CCD camera. One peak is red detuned, and the other peak is formed blue detuned to the atomic transition at *ω*_*a*_ as shown in Fig. 2(a). The power of light coming out of the cavity measured in the PMT is converted to the average number of photons in the cavity, $\bar{n}$ by the expression, $\bar{n}=\frac{{P}_{out}}{2\hslash {\omega }_{c}{\kappa }_{r}}$, where *P*_*out*_ is the power of light out of the cavity, *ω*_*c*_/2*π* is the frequency of cavity resonance, and 2*κ*_*r*_/(2*π*) is the measured rate of transmission from one of the cavity mirror. Both these peaks show TEM_00_ spatial mode structure imaged in the camera, i.e., the cavity output has Gaussian intensity profile. Other spatial modes also show similar two peaked behavior. The peak heights and frequencies of both the peaks depend on *N*_*c*_ and power in the MOT beams. The red detuned peak is only visible above a critical number of atoms (*N*_*c*_ ~ 20 × 10^3^) and grows rapidly to dominate the blue detuned peak for atom number *N*_*c*_ ~ 27 × 10^3^ as seen from the Fig. 2(c). In addition, from Fig. 2(a) the peaks are seen to broaden as *N*_*c*_ increases. It should be noted that the width of the red detuned peak is narrower than the blue detuned peak in all the measurements.

The dependence of the peak separation as a function of *N*_*c*_, when the cavity length is scanned across the atom-cavity resonances is illustrated in Fig. 2(b). The blue dots show corresponding vacuum Rabi splitting (VRS) calculated for the same number of atoms and cavity parameters when the cavity is on the atomic resonance, and external weak probe light scans the atom-cavity system. From this close agreement between the measured frequency split and the VRS calculation we can see that the observed split depends on ${g}_{0}\sqrt{{N}_{c}}$, similar to VRS, and hence we conclude that the observed splitting and the atom-cavity collective strong coupling are very closely related. However, this is the case only beyond a certain atom number in the cavity mode. Below a critical value of *N*_*c*_, the red-detuned peak disappears while the blue detuned peak persists, as seen in Fig. 2(c). Here a clear threshold is seen in the measured heights of the red detuned peak, while the blue detuned peak shows no threshold over the measurement range. It is also evident from Fig. 2(b) and (c) that the agreement with the VRS calculation starts degrading at the larger values of *N*_*c*_, which coincides with the tendency towards saturation of the red detuned peak height. The intensity of the MOT beams for these measurements was 36 mW/cm^2^.

**Figure 3:**
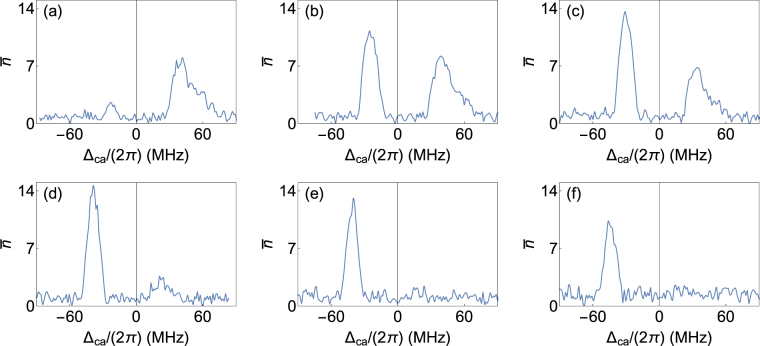
Change in emission from the cavity when total intensity of MOT laser is varied. Intensities and atoms coupled (*N*_*c*_) are (**a**) 22 mW/cm^2^ and (35 ± 1) × 10^3^, (**b**) 30 mW/cm^2^ and (33 ± 1) × 10^3^, (**c**) 36 mW/cm^2^ and (29 ± 1) × 10^3^, (**d**) 45 mW/cm^2^ and (24 ± 1) × 10^3^, (**e**) 60 mW/cm^2^ and (21 ± 1) × 10^3^, (**f**) 75 mW/cm^2^ and (23 ± 1) × 10^3^ respectively.

Given the qualitative difference in the nature of the red and blue detuned peaks, and the fact that the red detuned peak shows threshold behavior, indicative of lasing it is important to determine the effect of driving the MOT atoms deeper into saturation on the two peaks. For very low power the red detuned peak vanishes and when the power in MOT laser is high the blue detuned peak vanishes as can be seen in Fig. 3. Increasing the power increases total atom number but also increases the spatial size of the atomic cloud in MOT. The increase in size is attributed to an increase in temperature of the MOT atoms^35–37^. The increase in size lowers *N*_*c*_, and as fewer atoms overlap the cavity mode, the rate of fluorescence into the cavity decreases reducing the size of the blue detuned peak. Lower number of *N*_*c*_ should also result in reduction of red detuned peak according to Fig. 2(c). However, when the atoms are driven harder two factors play a role in higher amplitude of red detuned peak, (a) the relative fluorescence into the Mollow sidebands of the fluorescence spectrum increases and (b) the rate of stimulated emission for red detuned peak increases as will be clear in the section which discusses the gain mechanism for lasing in red detuned peak. However, once the lasing starts to saturate, the effects of the increase in the size of atomic cloud and reduction in *N*_*c*_ lead to a reduction in amplitude of red detuned peak as can be seen from Fig. 3(e) and (f). This provides experimental evidence of crucial differences between the blue and red detuned emission peaks.

### Signatures of lasing

**Figure 4:**
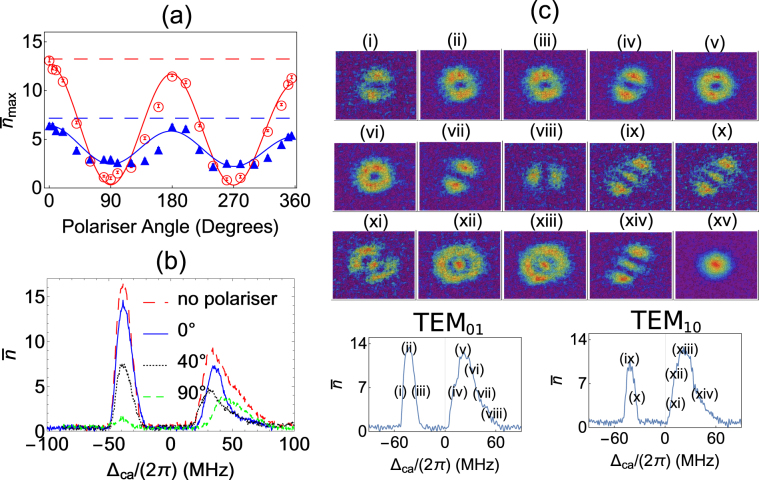
Mode purity measurements. (**a**) Polarization diagnosis of the light coming out of the cavity. Blue filled triangles denote the height of the blue detuned peak, and red empty circles denote the height of the red detuned peak. Red dashed and blue dashed are peak values when we do not have a polarizer in the cavity output path multiplied by transmission coefficient of the polarizer. The smooth curves are obtained by fitting cosine function multiplied by a linear function, $(a+b\,\cos \,[\frac{\pi }{180}x])(1-cx)$. The linear function is used to correct for the systematic effect of atom number decrease during long measurements. (**b**) Cavity output profile for different polarizer angles. Here, *N*_*c*_ = (26 ± 1) × 10^3^ for the above graphs. (**c**) Modes of the cavity as seen on a CCD camera at various detunings of the cavity. Lower case Roman numerals (i–viii) are for TEM_01_ mode and (ix–xiv) are for TEM_10_ mode. (xv) shows an instance of TEM_00_ mode. Here, for TEM_00_ mode *N*_*c*_ = (33 ± 1) × 10^3^, it will be lower for higher order modes. The power of MOT cooling laser is 36 mW/cm^2^ for all the cases.

The threshold behavior seen only in the red detuned peak in Fig. 2(c) is suggestive of lasing in this driven atomic system coupled to the cavity mode. In lasing one mode experiences large gain compared to others as stimulated emission ensures that the dominant mode wins. As a check for lasing by the atom-cavity system, we check for purity of the state of light that comes out of the cavity. The first check is for the polarization state of the cavity emitted light using a polarizer (Thorlabs LPNIRE100-B) with variable polarization angle in between the cavity mirror and the PMT. Figure 4(a) shows the change in peak height of both red and blue detuned peaks as the polarization angle is changed. The polarization of the red detuned peak is considerably purer than that of the blue detuned peak. From the fits to data, the visibility for the red detuned peak is around 95% and for the blue detuned peak is around 43%. The small systematic change in amplitude with polarizer angle is due to the slight decrease in the atom number as the measurement progressed. In addition, Fig. 4(b) shows that the shape of the blue detuned peak depends on polarizer angle. The peak and the wings of the blue detuned peak show different polarizations. This shows that the light out of the cavity for the red peak is strongly polarized and indicative of a single cavity mode present. On the other hand, the blue detuned peak has multiple polarization states in coexistence, which is indicative of no mode competition and therefore is detected as a mixture of several simultaneously existing polarization states. The consistent explanation for this observation is that the light out of the cavity for the red peak is lasing. All the data mentioned above was for TEM_00_ spatial mode as there are no nearby spatial modes within the cavity linewidth which will compete with the TEM_00_ spatial mode, the only competition is between the polarization states of the spatial mode. The existence of single spatial mode is verified by imaging the spatial mode of light emitted by the cavity. However, for higher spatial modes there is competition between the cylindrical and rectangular modes which are nearly degenerate as can be seen explicitly from Fig. 4(c). Again, similar to the polarization states, a single dominant spatial mode is observed for the red detuned peak while the blue detuned cavity emission peak shows multiple spatial modes of similar intensities. Experimentally, the purity of polarization and the spatial mode is strong evidence in support of lasing in the case of the red detuned peak. The sharp threshold for the red detuned peak and the rapid increase in peak height as the atom number is increased is characteristic of lasing behavior arising due to non-linear response with an increase in gain^38^.

**Figure 5:**
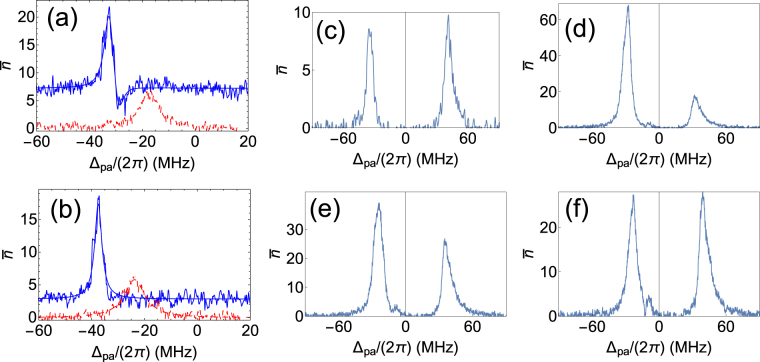
Probe transmission spectra when atoms are present inside the cavity. For (**a**) and (**b**), the blue trace shows cavity probe transmission when driven atoms are present in the cavity, and the broken red trace is probe transmission curve without atoms. (**a**) This figure shows gain and Fano-like profile in the presence of atoms. The cavity length is such that we are near the top of the red detuned peak of the last panel in Fig. 2(a). We can see gain in the probe at around 33 MHz. The blue curve in the background is fit to the sum of a Lorentzian and a inverted Lorentzian both of FWHM 4.5 MHz, and the red dashed curve for the empty cavity is a Lorentzian with FWHM of 9.5 MHz. (**b**) This figure shows line narrowing and gain without the Fano profile when the cavity is −5.5 MHz of the red detuned peak of the last panel in Fig. 2(a). A Lorentzian fit to the blue trace gives FWHM of 3.3 MHz and is shown as the blue curve. Here, for (**a**) and (**b**) *N*_*c*_ = (33 ± 1) × 10^3^. (**c**) Without MOT laser and cavity frequency same as atomic frequency. (**d**) With MOT laser and cavity frequency same as atomic frequency. (**e**) With MOT laser and cavity frequency towards blue of atomic frequency (**f**) With MOT laser and cavity frequency more towards blue of atomic frequency to make the heights of the peak same. For (**c**–**f**), *N*_*c*_ = (25 ± 1) × 10^3^. The power in MOT laser for all the measurements in this figure is 36 mW/cm^2^.

To determine the frequency at which the gain occurs, we pass a probe beam through the cavity and monitor the transmission. Figure 5(a) shows the transmitted intensity when the frequency of the probe beam is scanned while keeping the cavity detuning fixed on the red detuned peak of the last panel in Fig. 2(a). The blue trace represents the output with MOT atoms, and the red trace is the output when there are no atoms in MOT (the magnetic field of the MOT is switched off). As is evident from the differences in the transmission in Fig. 5, the probe beam experiences gain which is more than the total losses when interacting with atoms inside the cavity. The shift between blue and the red curve is due to lasing without any probe laser as we make sure that the cavity detuning is fixed on the red peak of the last panel in Fig. 2(a). In addition to gain, the blue trace also shows a Fano-like profile indicating interference between two or multiple probability amplitudes for emission^39^ which suppresses the lasing action. Figure 5(b) shows line narrowing even in the absence of Fano profile for cavity resonance 5.5 MHz red of the red detuned peak of the last panel in Fig. 2(a). A similar experiment on the blue detuned peak attenuates and shows a tendency to broaden the transmitted light.

### Gain mechanism and collective strong coupling

The phenomenon of gain in probe beam when two-level atoms are strongly driven by another drive laser is well known. B. R. Mollow was first to predict such gain mechanism^40^ using semi-classical arguments and phenomenologically including atomic decay rates. Haroche *et al*.^41^ reported similar calculations in the same year. This was later observed in an experiment with sodium beam by Wu *et al*.^30^. Grynberg *et al*.^29^ later proposed a microscopic description for gain in the above experiments and calculated the cross-section of various relevant multiphoton processes using perturbation theory. For this, they analyzed processes involving spontaneous emission of one and two photons. For the parameters they considered, out of all the photon scattering processes in which only one photon is spontaneously emitted, the scattering processes leading to absorption of the probe photon dominates. Hence, single photon emission processes always lead to net absorption of the probe photon. They then showed that processes resonant in dressed state picture which involve spontaneous emission of two photons result in both absorption and amplification of the probe beam. However, these have equal cross-sections and hence cancel out. This balance is changed by interference between resonant and non-resonant absorption probability amplitudes involving emission of two spontaneous emission photons. This interference leads to a term in the cross section for absorption proportional to $|{\rm{\Omega }}{|}^{4}({\omega }_{d}-{\omega }_{p})/{{\rm{\Delta }}}_{da}^{3}$, where ${\omega }_{p}\mathrm{/(2}\pi )$ is frequency of the probe laser, ${\omega }_{d}\mathrm{/(2}\pi )$ is frequency of the drive laser, ${{\rm{\Delta }}}_{da}=({\omega }_{d}-{\omega }_{a})$ is detuning of drive laser from the atomic transition, and $2{\rm{\Omega }}\mathrm{/(2}\pi )$ is Rabi frequency of the drive laser. For ${{\rm{\Delta }}}_{da}({\omega }_{d}-{\omega }_{p}) < 0$ this interference term will have a negative sign and hence will reduce the absorption cross section for the probe photon. In this case, the non-resonant processes leading to probe photon amplification were negligible compared to non-resonant processes leading to probe photon absorption hence was neglected resulting in no change in the amplification cross section. This decrease in absorption for a probe photon can lead to net amplification which is the source of gain in such systems. Additionally, this reduction in absorption is proportional to $|{\rm{\Omega }}{|}^{4}$, i.e. $\propto {I}_{d}^{2}$, where *I*_*d*_ is the intensity of the drive beam, which explains the rapid increase in red detuned peak in Fig. 3. The mechanism for the gain in our experiment is similar to the description of Grynberg *et al*.^29^, with the additional consideration that the cavity-atom collective strong coupling regime introduces certain differences discussed below.

In the regime defined by collective strong coupling, weak probe transmission through the cavity is split into two peaks when resonant atoms are present in the cavity due to VRS^3, 7, 14, 34^. Figure 5(c) is a measurement of such a split for which the MOT lasers were switched off, and all the atoms were in the ground state^12^. Here, if the cavity resonance frequency is matched with the atomic frequency we see symmetric splitting on the red and the blue side of the atomic resonance. However, if the cavity resonance is blue (red) detuned with respect to the atomic frequency the blue (red) detuned VRS peak is bigger than red (blue) detuned one^34^ and blue (red) peak shifts away (towards) from atomic frequency. However, when the drive laser with a frequency different from atomic transition is switched on, symmetry is broken because of unequal gain and loss. Figure 5(d,e,f) shows such a scenario. For Fig. 5(d), when the MOT drive lasers are present, the frequency spectrum remains unchanged, but the heights are completely different, indicating an unequal total loss. The drive (MOT cooling laser) is to the red side of atomic frequency. For Fig. 5(e) even though the cavity resonance is set to the blue of the atomic resonance, the red detuned peak is dominant. In the absence of drive laser, the height of blue detuned peak would have increased, and the height of red detuned peak would have diminished. However, due to Mollow gain in the the red detuned probe and Mollow loss in the blue detuned probe in the presence of the red detuned drive we see the opposite in Fig. 5(e) with respect to what is expected in the case when the atoms are not optically driven. Equal height for the two peaks is obtained when the cavity resonance is 8 MHz detuned towards blue of atomic frequency as shown in Fig. 5(f). In addition to this, there also seems to be gain around −13 MHz which is the drive frequency. This is because of coherent energy exchange between drive and cavity. The observations mentioned above are compared with the results of a semi-classical theory in the discussion section.

In addition to gain in the probe, we also observe lasing without any seeding (no probe light). This is because of the coupling of light into the cavity by the fluorescing atoms. B. R. Mollow was first to calculate the fluorescence spectrum of a two-level atom driven by a strong monochromatic laser^42^. Such a spectrum can be computed from fluctuations of atomic coherence^43^ and is shown in the Supplementary Fig. S1. There is some light at ~30 MHz red of the atomic transition. This resonance fluorescence provides the seed which is amplified by the driven atoms.

## Discussion

The interpretation of the observed gain rests on the theoretical work of B. R. Mollow^40^ and Grynberg *et al*.^29^ which was for a free-space probe beam and hence their theoretical modeling did not include a cavity. Additionally, Grynberg *et al*.^29^ in their full quantum calculations assumed $\mathrm{4|}{\rm{\Omega }}/{{\rm{\Delta }}}_{da}{|}^{2}\ll 1$ in order to perform the calculations using perturbation theory. In our experiment, $\mathrm{4|}{\rm{\Omega }}/{{\rm{\Delta }}}_{da}{|}^{2} \sim 1$ hence the perturbation approach is not possible, and a full quantum calculation is very challenging. Hence, in order to quantitatively compare our experiment with a theory, we modeled our experimental system using the semi-classical theory of light-matter interactions adapted from the calculations of B. R. Mollow^40^. The model contains all the essential elements required to show gain consistent with the experimental observations. We consider 2-level atoms coupled to one of the modes of a Fabry-Pérot cavity, which is probed by a monochromatic laser of frequency *ω*_*p*_ and the atoms are driven by a single drive laser with frequency *ω*_*d*_. The probe and drive light fields are assumed to be classical. The calculation is provided in the Supplementary Material.

**Figure 6:**
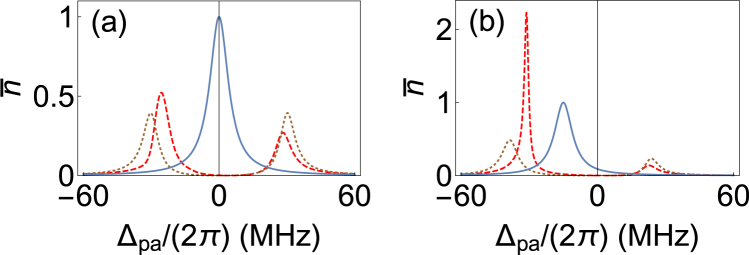
Probe transmission through the cavity as a function of detuning from the atomic transition. Blue is empty cavity transmission, the dotted brown curve is the VRS, and red dashed curve shows probe transmission when atoms are driven by classical light. The two graphs are for different detuning of the cavity from atomic transition, (**a**) ${{\rm{\Delta }}}_{ca}\mathrm{/(2}\pi )=0$ and (**b**) ${{\rm{\Delta }}}_{ca}\mathrm{/(2}\pi )=-15$ MHz. Other parameters are strength of drive field, ${\rm{\Omega }}\mathrm{/(2}\pi )=6$ MHz, strength of probe field $\eta \mathrm{/(2}\pi )=4.75$ MHz, atom-cavity collective strong coupling, ${g}_{t}\mathrm{/(2}\pi )=30$ MHz, ${\rm{\Gamma }}\mathrm{/(2}\pi )=6$ MHz, cavity FWHM, $2\kappa \mathrm{/(2}\pi )=9.5$ MHz, and ${{\rm{\Delta }}}_{pa}\mathrm{/(2}\pi )=-13$ MHz. $\bar{n}$ is the average intracavity photon number.

The average photon number in the cavity can be calculated using the semi-classical theory mentioned above. Figure 6 shows cavity transmission spectrum as a function of the frequency of probe laser using Supplementary Eqn. S16. From Fig. 6 it is evident that for red detuning the probe laser experiences gain and for blue detuning experiences loss. This is completely consistent with the experimental observations in Fig. 5. When the gain of probe laser from atoms exceeds the loss from the atom-cavity system, we see lasing as shown in Fig. 6(b) providing a consistency check for the lasing of the red-detuned peak. In addition, the parameters in our calculations are such that $\mathrm{4|}{\rm{\Omega }}{|}^{4} < |{{\rm{\Delta }}}_{da}{|}^{3}{\rm{\Gamma }}$ and $\mathrm{4|}{\rm{\Omega }}{|}^{2} < |{{\rm{\Delta }}}_{da}{|}^{2}$, where ${\rm{\Gamma }}\mathrm{/(2}\pi )$ is decay rate of the atomic excited state due to spontaneous emission. This is opposite to the conditions necessary for gain in probe beam according to the theoretical calculations of Grynberg *et al*.^29^ and B. R. Mollow^40^ respectively, where the system was required to be driven hard. However, here the presence of cavity enhances the interaction, and we see gain even without the very demanding requirements on parameters in the earlier experiments.

On changing the detuning of the cavity with respect to the atomic transition, the collective eigenstate (VRS) also shifts in frequency. When the cavity is blue detuned with respect to the atomic transition, the red VRS peak shifts towards the atomic transition and the blue peak shifts away from the atomic transition. This makes it possible for the red VRS peak to be in resonance with the MOT cooling laser which is 13 MHz red detuned from the atomic transition when the detuned cavity resonance is to the blue side. As can be seen from Supplementary Fig. S1, the atomic fluorescence is dominant at MOT/drive laser frequency. Hence, the combined system of atoms and cavity becomes resonant for most of the fluorescent light, when the cavity is blue detuned with respect to the atomic transition. This explains the observation of the blue peak in the experiment as shown in the Figs 2(a), 3 and 4(b). This blue peak is unpolarized as the fluorescence can be of any polarization.

Comparing the numbers in the above calculations and the experiment, the total intensity of all the MOT beams and dipole moment for isotropic beam case gives ${\rm{\Omega }}\mathrm{/(2}\pi )\approx 6$ MHz as an estimate. However, theoretical treatment is for single drive beam whereas there are six beams, all with different polarization and a non-zero relative phase difference between them. The calculation therefore is a lower estimate of the light-atom interaction, since sub-Doppler cooling mechanisms will try to localize atoms towards intensity maxima formed by the six MOT beams increasing effective value of Ω^37^. In addition, we have assumed the cavity field to be very weak, such that the atomic coherence, *ρ* is only first order in cavity field, *α*. There is a possibility of gain to be higher order in *α* which has not been considered here. Hence exact quantitative comparison is not possible with the present model, but qualitatively we do not expect the physics behind the observations to change.

In conclusion, when a MOT of actively driven atoms is in collective strong coupling regime with a Fabry-Pérot cavity, lasing is seen in the red detuned emitted light from the atom-cavity system. The lasing for a stabilized cavity and MOT atom number is continuous in nature. This driving field is the same as the MOT light which cools and traps the atoms and results in continuous operation of the laser from the coupled atom-cavity system. The lasing is seen for the red detuned peak because symmetry is broken in favor of the red detuned peak due to the MOT light, which also confines the atoms effectively and couples it to the cavity mode. In principle, the lasing could be achieved on the blue detuned peak if the driving frequency and the trap mechanism were not connected and the driving light was blue detuned with respect to the atomic transition. The observed phenomena are therefore generic to the driven atom-cavity system and have potential to be of interest for basic studies and for applications. To analyze the basic processes of this phenomena a semiclassical theory of light-matter interaction is presented in the Supplementary, where the role of Mollow gain and dispersion due to the collective strong coupling of atoms to the cavity and the change in the dynamics of the system is explained. Here, we took the decay of the atomic excited state phenomenologically, similar to B. R. Mollow^40^. Theoretically, it would be a useful exercise to perform a full quantum and multimode calculation including the vacuum modes similar to Grynberg *et al*.^29^. The vacuum modes and the drive field can be then integrated out similar to the approach of open quantum optics^20^ giving an effective non-linear interaction between the photons in the cavity and the atoms. Such an approach will provide more information regarding the properties of laser observed in our experiment. Future work can be taken up to observe the properties of this type of non-linear laser, such as photon statistics, phase diffusion and squeezing, as predicted by single mode theories of Zakrzewski *et al*.^44–46^ and Agarwal^47, 48^.

## Electronic supplementary material

Supplementary Material for Lasing by driven atoms-cavity system in collective strong coupling regime
