# Supplementary material for: Lasing by driven atoms-cavity system in collective strong coupling regime
Source: Sci Rep. 2017 Sep 12;7:11432. doi: 10.1038/s41598-017-11799-5 (PMC5595928; doi:10.1038/s41598-017-11799-5)
Supplement: Supplementary file 1 — Supplementary Material for Lasing by driven atoms-cavity system in collective strong coupling regime [file 41598_2017_11799_MOESM1_ESM.pdf]

Supplementary Material for
Lasing by driven atoms-cavity system in collective strong coupling regime

Rahul Sawant and S. A. Rangwala
Raman Research Institute, Sadashivanagar, Bangalore, India.

S.1. DETAILS OF CALCULATIONS

The Hamiltonian of a system of driven N two-level atoms interacting with a cavity driven by a probe laser can be written as,

$$\hat{H} = \hbar\omega_c\ \hat{a}^\dagger\hat{a} + \hbar\left\{\eta^*\hat{a}e^{-i\omega_p t} + \eta\hat{a}^\dagger e^{i\omega_p t}\right\} + \sum_j^N \hbar\left\{\frac{\omega_a}{2}\hat{\sigma}_j^z + g_j\left(\hat{a}^\dagger\hat{\sigma}_j^- + \hat{a}\hat{\sigma}_j^+\right)\right\} + \sum_j^N \hbar\left\{\Omega^*\hat{\sigma}_j^- e^{i\omega_d t} + \Omega\hat{\sigma}_j^+ e^{-i\omega_d t}\right\}. \quad \text{(S1)}$$

$\hat{a}$ and $\hat{a}^\dagger$ are photon annihilation and creation operators for the cavity field with commutation relation $[\hat{a}, \hat{a}^\dagger] = 1$. σ_j^-, σ_j^+, σ_j^z are usual spin-$\frac{1}{2}$ Pauli operators for the jth atom with commutation relations $[\sigma_j^+, \sigma_j^-] = \sigma_j^z$ and $[\sigma_j^z, \sigma_j^\pm] = \pm\sigma_j^\pm$. $g_j = g_0 f(x_j, y_j)$ is coupling of jth atom with the cavity mode with $g_0 = -\mu\sqrt{\omega_c/(2\hbar\epsilon_0 V)}$ maximum atom-cavity coupling, $f(x, y) = e^{-(x^2+y^2)/w_0^2}$ is mode function of the cavity field, $V = \pi w_0^2 L_c/4$ is the mode volume of the cavity, L_c is cavity length, μ is the transition dipole matrix element between excited and ground state, $2|\Omega|/(2\pi) = \frac{\mu}{2\hbar\pi}\sqrt{\frac{2I_d}{c\epsilon_0}}$ is Rabi frequency for drive beam, I_d is intensity of the drive beam, η is strength of classical cavity probe. $\omega_c/(2\pi)$, $\omega_a/(2\pi)$, $\omega_d/(2\pi)$, $\omega_p/(2\pi)$ are frequencies of the cavity (resonance frequency of cavity when it is empty), atom, driving laser and cavity probe respectively and * denotes complex conjugate.

S.1.1. Rate equations

The evolution equation for the expectation value for an operator $\hat{X}$ can be evaluated using the Heisenberg equation, $\frac{d\langle\hat{X}\rangle}{dt} = \frac{i}{\hbar}\langle[\hat{H}, \hat{X}]\rangle$. In addition to this unitary evolution, we introduce non-unitary decay rates κ and Γ phenomenologically for cavity field and atoms respectively. This approach is similar to the one taken by B. R. Mollow [1]. For the calculation here, the cavity field is assumed to be classical and is denoted by a coherent state $|\alpha\rangle$. Using the Heisenberg equation and decay rates mentioned above for system variables give,

$$\frac{d\alpha(t)}{dt} = -\kappa\alpha(t) - i\omega_c\alpha(t) - i\sum_j^N g_j\rho_j(t) - \eta e^{-i\omega_p t} \quad \text{(S2a)}$$

$$\frac{d\rho_j(t)}{dt} = -\left\{\frac{\Gamma}{2} + i\omega_a\right\}\rho_j(t) + i(g_j\alpha(t) + \Omega e^{-i\omega_d t})(2\rho_j^e(t) - 1) \quad \text{(S2b)}$$

$$\frac{d\rho_{e,j}(t)}{dt} = -\Gamma\rho_{e,j}(t) + i\left\{(g_j\alpha^*(t) + \Omega^* e^{i\omega_d t})\rho_j(t) - g_j\alpha(t) + \Omega e^{-i\omega_d t})\rho_j^*(t)\right\} \quad \text{(S2c)}$$

$\rho_j = \langle\sigma_j^-\rangle$ is coherence of the jth atom, $\alpha = \langle\hat{a}\rangle$ is field amplitude inside the cavity and $\rho_{e,j} = \langle\sigma_{e,j}\rangle$ is excited state population.

As multiple frequencies are involved, we transform the time-dependent equations (2) into Fourier space. For the cavity field and the atomic coherences, the Fourier transforms are, $\alpha(t) = \int_{-\infty}^{\infty}\tilde{\alpha}(\omega)e^{-i\omega t}d\omega$ and $\rho_j(t) = \int_{-\infty}^{\infty}\tilde{\rho}_j(\omega)e^{-i\omega t}d\omega$ respectively. And as $\rho_{e,j}(t)$ is always real, its Fourier transform takes the form, $\rho_{e,j}(t) = \int_{-\infty}^{\infty}\frac{1}{2}(\tilde{\rho}_{e,j}(\omega)e^{-i\omega t} + \tilde{\rho}_{e,j}^*(\omega)e^{i\omega t})d\omega$ with the relation $\tilde{\rho}_{e,j}^*(-\omega) = \tilde{\rho}_{e,j}(\omega)$. This relation can be verified by replacing ω with $-\omega$ in Fourier relation of $\rho_{e,j}(t)$. The inverse transforms are, $\tilde{\alpha}(\omega) = \int_{-\infty}^{\infty}\alpha(t)e^{-i\omega t}dt$, $\tilde{\rho}_j(\omega) = \int_{-\infty}^{\infty}\rho_j(t)e^{i\omega t}dt$. $\tilde{\rho}_{e,j}(\omega) = \int_{-\infty}^{\infty}\rho_{e,j}(t)e^{i\omega t}dt$ and $\tilde{\rho}_{e,j}^*(\omega) = \int_{-\infty}^{\infty}\rho_{e,j}(t)e^{-i\omega t}dt$.

This gives a set of equations,

$$-i\omega\tilde{\alpha}(\omega) = -\kappa\tilde{\alpha}(\omega) - i\omega_c\tilde{\alpha}(\omega) - i\sum_j^N g_j\tilde{\rho}_j(\omega) - \eta\delta(\omega-\omega_p) \tag{S3a}$$

$$-i\omega\tilde{\rho}_j(\omega) = -\left\{\frac{\Gamma}{2} + i\omega_a\right\}\tilde{\rho}_j(\omega) + ig_j\left\{2(\tilde{\alpha}\odot\tilde{\rho}^*_{e,j})(\omega)_- - \tilde{\alpha}(\omega)\right\} + i\Omega\left\{2\tilde{\rho}^*_{e,j}(\omega_d-\omega) - \delta(\omega-\omega_d)\right\} \tag{S3b}$$

$$i\omega\tilde{\rho}^*_{e,j}(\omega) = -\Gamma\tilde{\rho}^*_{e,j}(\omega) + ig_j\left\{(\tilde{\alpha}^*\odot\tilde{\rho}_j)(\omega)_- - (\tilde{\alpha}\odot\tilde{\rho}^*_j)(\omega)_+\right\} + i\left\{\Omega^*\tilde{\rho}_j(\omega_d-\omega) - \Omega\tilde{\rho}^*_j(\omega_d+\omega)\right\} \tag{S3c}$$

Here, eqn (2a) and eqn (2b) are multiplied by $e^{i\omega t}$ and eqn (2c) by $e^{-i\omega t}$ and then integration $\int_{-\infty}^{\infty} dt$ is done. $(\tilde{\alpha}\odot\tilde{\rho}^*_j)(\omega)_\pm = \int\tilde{\alpha}(\omega_1)\tilde{\rho}^*_j(\omega_1\pm\omega)d\omega_1$ are convolution functions where $\odot$ denotes convolution operation. δ is Dirac delta function.

To understand the physics we discuss the various interactions sequentially. First, we neglect the classical driving of the atoms, i.e. $\Omega = 0$ and assume that the cavity field (probe laser) is so weak that the atoms remain mostly in ground state, i.e $\tilde{\rho}_{e,j}(\omega) = \tilde{\rho}^*_{e,j}(\omega) \approx 0$. For such a case the cavity field is,

$$\tilde{\alpha}(\omega_d) = \frac{-\eta\left\{\frac{\Gamma}{2} - i\Delta_{pa}\right\}}{\{\kappa - i\Delta_{pc}\}\left\{\frac{\Gamma}{2} - i\Delta_{pa}\right\} + g_t^2} \tag{S4}$$

$g_t = \sqrt{\sum_j^N g_j^2}$ is total g for all the atoms and is equal to $g_0\sqrt{N_c}$, where g_0 is the single atom-cavity mode coupling and N_c is an average number of atoms coupled to the cavity and can be calculated using an overlap of the cavity mode and the atomic density distribution. $\Delta_{pc} = \omega_p - \omega_c$ and $\Delta_{pa} = \omega_p - \omega_a$ are detunings of the cavity probe laser from the cavity and the atomic frequencies respectively. Eqn. (4) gives the usual splitting in the cavity peak (VRS/normal mode splitting). The splitting between two peaks is equal to $2g_t = 2g_0\sqrt{N_c}$.

Now, in the absence of a cavity all the atoms behave in same way. This is because all are driven equally by the classical drive field. The atomic variables in this case are,

$$\tilde{\rho}(\omega_d) = \frac{-2i\Omega(2i\Delta_{da}+\Gamma)}{\Gamma^2 + 4\Delta_{da}^2 + 8|\Omega|^2} \quad \text{and} \quad \tilde{\rho}_e(0) = \frac{4|\Omega|^2}{\Gamma^2 + 4\Delta_{da}^2 + 8|\Omega|^2} \tag{S5}$$

$\Delta_{da} = \omega_d - \omega_a$ is detuning of drive laser from the atomic transition. The j subscript is removed as the atom-field interaction strength is same for all the atoms. The atomic coherence oscillates only at frequency ω_d and all other frequency components are zero.

S.1.2. Perturbative calculation

For our experiment, $\Omega^2 \gg g_0^2|\alpha|^2$ and hence the effect of the cavity on the atoms is very small and adds only small perturbations to values in (5). The perturbed atomic variables can be written as, $\tilde{\rho}_j(\omega) = \tilde{\rho}(\omega_d)\delta(\omega-\omega_d) + \tilde{\epsilon}_j(\omega)$, $\tilde{\rho}_{e,j}(\omega) = \tilde{\rho}_e(0)\delta(\omega) + \tilde{\epsilon}_{e,j}(\omega)$and $\tilde{\rho}^*_{e,j}(\omega) = \tilde{\rho}_e(0)\delta(\omega) + \tilde{\epsilon}^*_{e,j}(\omega)$. Here, ϵ is perturbation to density matrix elements of atoms due to interaction with the cavity. Keeping only the unperturbed part in the convolution functions gives a set of linear equations,

$$-i\omega\tilde{\alpha}(\omega) = -\kappa\tilde{\alpha}(\omega) - i\omega_c\tilde{\alpha}(\omega) - i\sum_j^N g_j\tilde{\rho}_j(\omega) - \eta\delta(\omega-\omega_p) \tag{S6a}$$

$$-i\omega\tilde{\rho}_j(\omega) = -\left\{\frac{\Gamma}{2} + i\omega_a\right\}\tilde{\rho}_j(\omega) + ig_j\tilde{\alpha}(\omega)\left\{2\tilde{\rho}_e(0) - 1\right\} + i\Omega\left\{2\tilde{\rho}^*_{e,j}(\omega_d-\omega) - \delta(\omega-\omega_d)\right\} \tag{S6b}$$

$$i\omega\tilde{\rho}^*_{e,j}(\omega) = -\Gamma\tilde{\rho}^*_{e,j}(\omega) + ig_j\left\{\tilde{\alpha}^*(\omega_d+\omega)\tilde{\rho}(\omega_d) - \tilde{\alpha}(\omega_d-\omega)\tilde{\rho}^*(\omega_d)\right\} + i\left\{\Omega^*\tilde{\rho}_j(\omega_d-\omega) - \Omega\tilde{\rho}^*_j(\omega_d+\omega)\right\} \tag{S6c}$$

In zeroth order of the atomic terms, the cavity field gets an additional term,

$$i\sum_j^N g_j\tilde{\rho}_j(\omega) = i\sum_j^N g_j\left\{\tilde{\rho}(\omega_d)\delta(\omega-\omega_d) + \tilde{\epsilon}_j(\omega)\right\} \approx i\sum_j^N g_j\tilde{\rho}(\omega_d)\delta(\omega-\omega_d) \tag{S7}$$

This requires $\omega_p = \omega_d$ as seen in eqn (7) and results in an elastic exchange of energy from drive field to cavity field via atoms and the cavity will gain photons even if it is not driven by an external field.

For other frequencies the perturbation term $\tilde{\epsilon}_j(\omega)$ is important, hence we proceed to derive it using Eqn (6). Eqn (6b) can be rewritten as,

$$
\begin{aligned}
&-\left\{\frac{\Gamma}{2}+i\Delta_a(\omega)\right\}\tilde{\epsilon}_j(\omega)+ig_j\tilde{\alpha}(\omega)\left\{2\tilde{\rho}_e(0)-1\right\}+i\Omega\left\{2\tilde{\epsilon}^*_{e,j}(\omega_d-\omega)\right\}\\
&=\left(\left\{\frac{\Gamma}{2}+i\Delta_a(\omega)\right\}\tilde{\rho}(\omega_d)-i\Omega\left\{2\tilde{\rho}_e(0)-1\right\}\right)\delta(\omega-\omega_d)
\end{aligned}
\tag{S8}
$$

The right-hand side of above equation is zero for all ω because the term $\tilde{\rho}(\omega_d)$ was derived by putting the linear equation in right-hand side bracket with $\omega=\omega_d$ to zero in the absence of a cavity as can be inferred from Eqn (3) by putting the cavity field terms to zero. This gives,

$$
-\left\{\frac{\Gamma}{2}+i\Delta_a(\omega)\right\}\tilde{\epsilon}_j(\omega)+ig_j\tilde{\alpha}(\omega)\left\{2\tilde{\rho}_e(0)-1\right\}+i\Omega\left\{2\tilde{\epsilon}^*_{e,j}(\omega_d-\omega)\right\}=0 \tag{S9}
$$

Using (9) and $\tilde{\epsilon}^*_{e,j}(\omega_d-\omega)\approx 0$ gives first order term for $\tilde{\epsilon}_j(\omega)$,

$$
\tilde{\epsilon}_j(\omega_p)=-ig_j\frac{\tilde{\alpha}(\omega_p)\left\{-2\tilde{\rho}_e(0)+1\right\}}{\left\{\frac{\Gamma}{2}-i\Delta_{pa}\right\}}=-ig_j\tilde{\alpha}(\omega_p)C_1(\omega_p) \tag{S10}
$$

This is similar to the value of $\tilde{\rho}(\omega)$ used to calculate Eqn (4) but with an extra factor of $\{-2\tilde{\rho}_e(0)+1\}$. This factor comes because of change in population difference between excited and ground state and effectively reduces the coupling as the cavity field sees fewer atoms in the ground state.

Now for excited state population if we do not neglect the term, $\tilde{\epsilon}^*_{e,j}(\omega_d-\omega)$,

$$
\begin{aligned}
&(i\omega_d-i\omega+\Gamma)[\tilde{\rho}_e(0)\delta(\omega_d-\omega)+\tilde{\epsilon}^*_{e,j}(\omega_d-\omega)]\\
&=ig_j\left\{\tilde{\alpha}^*(2\omega_d-\omega)\tilde{\rho}(\omega_d)-\tilde{\alpha}(\omega)\tilde{\rho}^*(\omega_d)\right\}+i\left\{\Omega^*\tilde{\rho}_j(\omega)-\Omega\tilde{\rho}^*_j(2\omega_d-\omega)\right\}
\end{aligned}
\tag{S11}
$$

After rearranging we get,

$$
\begin{aligned}
&(i\omega_d-i\omega+\Gamma)\tilde{\epsilon}^*_{e,j}(\omega_d-\omega)-ig_j\left\{\tilde{\alpha}^*(2\omega_d-\omega)\tilde{\rho}(\omega_d)-\tilde{\alpha}(\omega)\tilde{\rho}^*(\omega_d)\right\}-i\left\{\Omega^*\tilde{\epsilon}_j(\omega)-\Omega\tilde{\epsilon}^*_j(2\omega_d-\omega)\right\}\\
&=\left[-(i\omega_d-i\omega+\Gamma)\tilde{\rho}_e(0)+i\left\{\Omega^*\tilde{\rho}_j(\omega_d)-\Omega\tilde{\rho}^*_j(\omega_d)\right\}\right]\delta(\omega_d-\omega)
\end{aligned}
\tag{S12}
$$

Again the right hand side is zero for all ω. The complete set of equations to calculate $\tilde{\epsilon}_j(\omega_p)$ at the cavity probe frequency can be derived from (9) and (12) and are given by,

$$
-\left\{\frac{\Gamma}{2}+i\Delta_a(\omega_p)\right\}\tilde{\epsilon}_j(\omega_p)+ig_j\tilde{\alpha}(\omega_p)\left\{2\tilde{\rho}_e(0)-1\right\}+i\Omega\left\{2\tilde{\epsilon}^*_{e,j}(\omega_d-\omega_p)\right\}=0 \tag{S13a}
$$

$$
-[i(\omega_d-\omega_p)+\Gamma]\tilde{\epsilon}^*_{e,j}(\omega_d-\omega_p)+ig_j\left\{\tilde{\alpha}^*(2\omega_d-\omega_p)\tilde{\rho}(\omega_d)-\tilde{\alpha}(\omega_p)\tilde{\rho}^*(\omega_d)\right\}+i\left\{\Omega^*\tilde{\epsilon}_j(\omega_p)-\Omega\tilde{\epsilon}^*_j(2\omega_d-\omega_p)\right\}=0 \tag{S13b}
$$

$$
-\left\{\frac{\Gamma}{2}+i\Delta_a(2\omega_d-\omega_p)\right\}\tilde{\epsilon}_j(2\omega_d-\omega_p)+ig_j\tilde{\alpha}(2\omega_d-\omega_p)\left\{2\tilde{\rho}_e(0)-1\right\}+i\Omega\left\{2\tilde{\epsilon}^*_{e,j}(\omega_p-\omega_d)\right\}=0 \tag{S13c}
$$

$$
-[i(\omega_p-\omega_d)+\Gamma]\tilde{\epsilon}^*_{e,j}(\omega_p-\omega_d)+ig_j\left\{\tilde{\alpha}^*(\omega_p)\tilde{\rho}(\omega_d)-\tilde{\alpha}(2\omega_d-\omega_p)\tilde{\rho}^*(\omega_d)\right\}+i\left\{\Omega^*\tilde{\epsilon}_j(2\omega_d-\omega)-\Omega\tilde{\epsilon}^*_j(\omega)\right\}=0 \tag{S13d}
$$

If the cavity is only driven at one frequency, ω_p, $\alpha(2\omega_p-\omega)=0$ in above equations. From (13) it can be seen that in addition to oscillating at the frequency ω_p the atomic coherence also oscillates at frequencies $\omega_p\pm\Delta_{pd}$, where $\Delta_{pd}=\omega_p-\omega_d$. This was an assumption made by B. R. Mollow in his article [1] for solving free space scenario of the same problem as ours. We arrive at it using Fourier transforms. Hence, for non-zero $\tilde{\epsilon}^*_{e,j}(\omega_d-\omega)$ we get,

$$
\tilde{\epsilon}_j(\omega_p)=-ig_j\tilde{\alpha}(\omega_p)[C_1(\omega_p)+C_2(\omega_p)] \tag{S14}
$$

Where $C_1(\omega_p)$ is same as in (10) and the value of $C_2(\omega_p)$ is,

$$
C_2(\omega_p)=\frac{-16|\Omega|^2(\Gamma-i\Delta_{dp})\left[\Gamma^2-2i\Gamma\Delta_{dp}+4\Delta_{pa}(\Delta_{pa}-\Delta_{dp})\right]}{(\Gamma-2i(\Delta_{pa}+\Delta_{dp}))\left(\Gamma^2+8|\Omega|^2+4\Delta_{pa}^2\right)\left[(\Gamma-i\Delta_{dp})\left(4\Delta_{pa}^2+(\Gamma-2i\Delta_{dp})^2\right)+8|\Omega|^2(\Gamma-2i\Delta_{dp})\right]} \tag{S15}
$$

Finally, $\tilde{\alpha}(\omega_p)$ takes the form,

$$
\tilde{\alpha}(\omega_p)=\frac{-\eta-i\tilde{\rho}(\omega_d)\delta(\omega_d-\omega_p)\sum_j^N g_j}{\{\kappa-i\Delta_{pc}\}+g_t^2[C_1(\omega_p)+C_2(\omega_p)]} \tag{S16}
$$

The average photon number in the cavity can now be easily calculated using, $\bar{n}=|\tilde{\alpha}(\omega_p)|^2$.

S.2. MOLLOW SPECTRUM

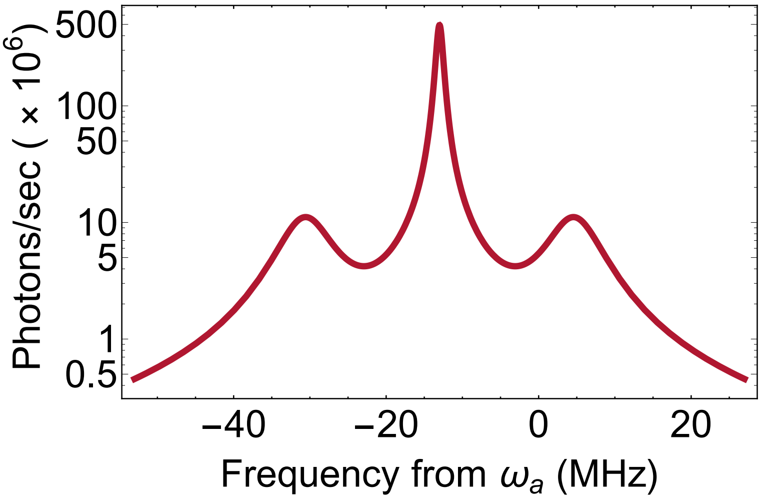

FIG. S1: Numerically computed Mollow spectrum on a log scale for parameters $\Omega/(2\pi) = 6$ MHz, $\Delta_{da}/(2\pi) = -13$ MHz, $N_c = 22 \times 10^3$, $\Gamma/(2\pi) = 6.06$ and MOT laser linewidth 1 MHz. Most of the light emitted is at MOT laser/drive frequency. y-axis shows the rate of photon emission into the cavity at that particular wavelength. This rate is upper bound on the actual rate because in the calculation of the rate we assume that all the atoms are at the centre of the cavity and hence all the light emitted by the atoms which fall on the cavity mirrors come back to the centre and form the cavity mode. Also, the emission from atoms is assumed to be isotropic. Hence the rate is $\zeta\times$ total rate of photon emission, where $\zeta = 0.009$ depends on the solid angle subtended by the mirrors at the centre of the cavity. The red sideband and blue sideband have centre frequencies -30.6 MHz $\approx (\Delta_{da} - \sqrt{4\Omega^2 + \Delta_{da}^2})/(2\pi)$ MHz and 4.6 MHz $\approx (\Delta_{da} + \sqrt{4\Omega^2 + \Delta_{da}^2})/(2\pi)$ respectively.

---

[1] B. R. Mollow, Physical Review A **5**, 2217 (1972), ISSN 0556-2791.
